# Association of Birth Order with Cardiovascular Disease Risk Factors in Young Adulthood: A Study of One Million Swedish Men

**DOI:** 10.1371/journal.pone.0063361

**Published:** 2013-05-16

**Authors:** Aline Jelenkovic, Karri Silventoinen, Per Tynelius, Mikko Myrskylä, Finn Rasmussen

**Affiliations:** 1 Department of Genetics, Physical Anthropology and Animal Physiology, University of the Basque Country Universidad del País Vasco/Euskal Herriko Unibertsitatea, Leioa, Spain; 1 IKERBASQUE, Basque Foundation for Science, Bilbao, Spain; 3 Department of Public Health, Hjelt Institute, University of Helsinki, Helsinki, Finland; 4 Population Research Unit, Department of Social Research, University of Helsinki, Helsinki, Finland; 5 Child and Adolescent Public Health Epidemiology, Department of Public Health Sciences, Karolinska Institutet, Stockholm, Sweden; 6 Max Planck Institute for Demographic Research, Rostock, Germany; Universidad Europea de Madrid, Spain

## Abstract

**Background:**

Birth order has been suggested to be linked to several cardiovascular disease (CVD) risk factors, but the evidence is still inconsistent. We aim to determine the associations of birth order with body mass index (BMI), muscle strength and blood pressure. Further we will analyse whether these relationships are affected by family characteristics.

**Methods:**

BMI, elbow flexion, hand grip and knee extension strength and systolic and diastolic blood pressure were measured at conscription examination in 1 065 710 Swedish young men born between 1951 and 1975. The data were analysed using linear multivariate and fixed effects regression models; the latter compare siblings and account for genetic and social factors shared by brothers.

**Results:**

Fixed effect regression analysis showed that birth order was inversely associated with BMI: second and third born had 0.8% and 1.1% (p<0.001) lower BMI than first-born, respectively. The association pattern differed among muscle strengths. After adjustment for BMI, first-born presented lower elbow flexion and hand grip strength than second-born (−5.9 N and −3.8 N, respectively, p<0.001). Knee extension strength was inversely related to birth order though not always significantly. The association between birth order and blood pressure was not significant.

**Conclusions:**

Birth order is negatively associated with BMI and knee extension strength, positively with elbow flexion and hand grip strength, and is not associated with blood pressure among young men. Although the effects are small, the link between birth order and some CVD risk factors is already detectable in young adulthood.

## Introduction

Cardiovascular diseases (CVD) are the leading cause of mortality worldwide and thus a major public health problem [1]. Obesity and hypertension are among the most important risk factors for CVD [2]–[4]. Previous studies have also shown that skeletal muscle strength, which is highly related to muscle mass, is inversely associated with the incidence of CVD [5]–[9]. CVD risk factors may, in turn, be influenced by several modifiable and non-modifiable secondary risk factors. Non-modifiable risk factors can include, but are not limited to, age, sex, ethnicity and some life history characteristics such as birth order, family size and maternal age at birth.

Birth order has been shown to be associated with several CVD risk factors in childhood, adolescence and young adulthood [10]–[17]. The mechanisms underlying these associations are, however, unclear and seem to be largely affected by the social, cultural and biological context [18]. It has been suggested that the lower birth weight observed in first-born implies a tendency to infant catch-up growth [19], which has been in turn associated with adverse metabolic and cardiovascular profile [20]–[24]. However, although the most common finding across studies indicates that first-borns face more disadvantageous levels of CVD risk factors, the evidence is still inconsistent. Birth order was inversely related to BMI in young men [13] and women [12]; however, positive [25] and non-significant [14], [26] relationships have also been reported. Increased adiposity has been associated with first-born status [11]–[13], but two recent studies detected that apart from the only children status, the last-born child presents an elevated risk of overweight and obesity in childhood [15], [16]. The birth order effect on blood pressure has also shown divergent results, with non-significant [13], negative [10], [14], [17] and J-shaped associations [27]. Finally, despite the beneficial role of muscular fitness in the prevention of diseases [28], whether muscle strength is influenced by birth order has not been studied yet.

The decline of fertility rate during the recent decades is decreasing the family size and consequently increasing the proportion of first born status in many countries [29]. Although birth order cannot be altered, identification of its impact is relevant for developing prevention and treatment strategies toward individuals at high risk. Accordingly, the aims of the present study are a) to assess the association of birth order with BMI, muscle strength and blood pressure and b) to investigate whether these relationships are affected by family characteristics in a large population of young Swedish men.

## Materials and Methods

### Ethics Statement

This study has been approved by the Ethical Review Board, Stockholm, Sweden. According to the current regulations, the Ethical Review Board waived the need for informed consent from the participants because this is a large register-based study without need to contact the participants.

### Data Collection

This longitudinal dataset was created by a record-linkage between the nationwide Swedish Military Service Conscription Register (MSCR), the Swedish Multi-Generation Register (MGR) and the Swedish Population and Housing Censuses (PHCs) using personal identification numbers. Conscription examination, which predates active military service, was mandatory in Sweden by law for all young male Swedish citizens in our study cohorts. Only males with severe handicap or a chronic disease were exempted from the conscription examination. In this study, we analysed cohorts born from 1951 to 1975 (conscription year 1969–1993). In the entire data set, we had conscription data available for 1 133 812 men. We excluded all multiple births (1.7% of the data). In addition, to keep the sample age-homogenous, men aged less than 17 or more than 20 years at conscription were excluded (18 027 men) since they represented only a small fraction of the whole study population (1.6%).

During the conscription examination, height, weight, elbow flexion, hand grip and knee extension strength, and diastolic (DBP) and systolic blood pressure (SBP) were measured according to a standardized protocol described elsewhere [30]. The measurement protocol of the muscle strength measures was not revealed to us by the Swedish Army. However, there were no systematic differences evident in the mean values of the measures between conscription offices, suggesting that a uniform protocol was used. The values of elbow flexion, hand grip and knee extension strength in these data were also close to values in a previous study of 31- to 35-year-old Finnish men [31]. Strength measures varied from 50 to 999 N, which was the maximum value the test could measure even if a participant was stronger. For knee extension this may create a minor ceiling effect because 0.13% of the participants reached the maximum value, whereas for elbow flexion and hand grip this proportion was smaller (0.06 and <0.01%, respectively). We had 16 051 cases (1.4% of the data) of missing or extreme values for height (<150 or >210 cm), weight (<40 or >150 kg), and BMI (<15 or >60 kg/m^2^). These values may have been true values or may represent measurement or data entry errors. To minimize errors of misclassification, we excluded these men from further analyses. In addition, we had missing values for muscle strength in 841 men. For blood pressure, the limits for accepted values were 40 to 100 mmHg for DBP and 100 to 180 mm Hg for SBP, with missing or invalid cases in 13641 men. In the final dataset, we had valid measures from all anthropometric and blood pressure traits on 1 065 710 men. Since BMI was not normally distributed, logarithmic transformation was applied. Information about conscription age and conscription centre was obtained from the MSCR. Based on continuous data in the MGR we created categorical variables for birth order (1, 2, …, 6+), maternal age at birth (5-year groups from 15–19 to 45–49 years) and number of children in the family (1, 2, …, 6+). Biological sisters were also taken into account for the calculation of birth order and family size. Information on parental education and occupational socioeconomic position (SEP) was derived from the PHCs conducted in 1960, 1970, 1980 and 1990 as described in detail elsewhere [32].

### Statistical Analyses

To study the association between birth order and CVD risk factors, linear regression analyses adjusted for different covariates were performed. Since BMI was log-transformed, the estimated regression coefficients for this variable can be interpreted as percentage changes (logBMI*100 =  % change). Model 1 adjusted for birth year, conscription age and centre. Model 2 added controls for maternal age and parental SEP and education. We continued to analyse within family associations by using fixed effects regression models (Model 3 and 4). These models compare brothers born to the same mother and remove the confounding influences of all fixed observed and unobserved genetic and social characteristics shared by the brothers [33]. Importantly, the fixed effects approach does not remove the potential confounding influence on non-shared factors. Model 3 was adjusted for the same covariates as those included in Model 1, and in Model 4 only maternal age was added, because fixed effects already controls for parental SEP and education. Moreover, since body size is a well-recognized factor that affects muscle strength [34], CVD risk factors were additionally adjusted for height (Model 5) and BMI (Model 6). This adjustment takes into account the effect of body size or mass, and thus allows to analyse the body size/mass-independent association between birth order and CVD risk factors. Confidence intervals and p-values were adjusted for clustering of brothers within families and were estimated using Stata/IC 12.0 (StataCorp, College Station, Texas, USA).

## Results

### Characteristics of the Participants

The characteristics of the sample are reported separately by birth order in Table 1. Some trends were detected across birth cohorts, in such a way that in more recent ones average number of children in the family was lower, there was a greater proportion of high parental SEP and education, and individuals were taller and heavier (results not shown). In Table 1 we summarize the mean values for all men, according to their birth order, as an average for all birth years. Mean age at conscription (18.3 years) did not differ among birth order groups, and as expected, average maternal age increased with birth order, from 24.4 years (1^st^ born) to 36.6 years (6^th^+born). Higher birth order was associated with older cohorts, larger families and lower proportion of high parental SEP (non-manual workers at higher and middle level) and education (more than 13 years). Regarding anthropometric and blood pressure traits, from birth order 1 to 6+ height and weight showed an average decrease of 1.7 cm and 1.4 kg respectively, whereas BMI remained stable. For all three muscle strength measures, second born presented the greatest mean values. For elbow flexion and hand grip strength, no defined pattern was observed. For knee extension strength, average value decreased monotonically with birth order (570.7 N to 540.3 N for 2^nd^ and 6^th^+born, respectively). The trends for SBP and DBP differed: whereas SBP showed the greatest mean values for birth order 1 and 6+, DBP increased monotonically from the second born.

**Table 1:** Subjects characteristics according to birth order.

	All orders Mean (SD)	1^st^ born Mean (SD)	2^nd^ born Mean (SD)	3^rd^ born Mean (SD)	4^th^ born Mean (SD)	5^th^ born Mean (SD)	6^th^+born Mean (SD)
*Socio-demographic*							
Conscription age (years)	18.3 (0.5)	18.3 (0.5)	18.3 (0.5)	18.3 (0.6)	18.3 (0.5)	18.3 (0.5)	18.3 (0.5)
Birth year	1963.0 (7.3)	1963.3 (7.3)	1963.3 (7.3)	1962.5 (7.2)	1961.5 (6.89)	1960.7 (6.65)	1959.9 (6.45)
Family size (number of children)	2.6 (1.2)	2.2 (0.9)	2.5 (0.8)	3.3 (0.9)	4.1 (1.0)	5.0 (1.0)	5.7 (0.9)
Maternal age (years)	27.3 (5.73)	24.4 (4.81)	27.7 (4.8)	30.6 (5.0)	32.9 (5.08)	34.5 (4.7)	36.6 (4.69)
High SEP (%) fathers/mothers	32.1/18.6	35.0/22.1	33.92/19.0	28.6/14.6	20.9/9.4	14.3/5.6	8.6/3.0
High education (%) fathers/mothers	14.7/14.6	16.7/17.5	15.2/14.7	12.9/11.4	9.0/7.1	5.1/3.9	2.3/1.8
*Anthropometrics and blood pressure*							
Height (cm)	179.2 (6.5)	179.4 (6.5)	179.3 (6.5)	179.0 (6.5)	178.5 (6.55)	178.1 (6.4)	177.5 (6.5)
Weight (kg)	69.5 (10.3)	69.7 (10.2)	69.5 (10.1)	69.3 (10.4)	69.1 (10.7)	68.6 (10.8)	68.1(10.6)
BMI (kg/m^2^)	21.62 (2.81)	21.65 (2.81)	21.59 (2.76)	21.62 (2.85)	21.64 (2.93)	21.59 (2.99)	21.59 (2.96)
Elbow flexion strength (N)	387.4 (84.3)	385.1 (84.1)	389.3 (84.4)	388.7 (84.4)	388.9 (84.8)	387.8 (84.3)	388.6 (83.4)
Hand grip strength (N)	616.3 (97.7)	614.4 (97.8)	618.4 (97.1)	617.2 (98.0)	616.3 (97.7)	616.7 (98.0)	615.7 (99.1)
Knee extension strength (N)	567.3 (117.3)	569.0 (117.6)	570.7 (117.2)	564.1 (117.1)	556.1 (116.2)	548.4 (114.4)	540.3 (112.7)
SBP (mmHg)	128.4 (10.8)	128.6 (10.9)	128.4 (10.8)	128.3 (10.8)	128.3 (10.9)	128.3 (10.7)	128.6 (11.0)
DBP (mmHg)	67.4 (10.0)	67.4 (10.0)	67.3 (9.9)	67.5 (9.9)	67.8 (9.9)	68.0 (10.0)	68.5 (10.0)
N of observations	1 065 710	450 151	364 761	156 753	56 332	21 220	16 943

Mean values and (standard deviations).

BMI, body mass index; DBP, diastolic blood pressure; SBP, systolic blood pressure; SD, standard deviation.

### Regression Analysis

Table 2 shows the linear regression analyses assessing the birth order effect on CVD risk factors. Model 1 presents the results adjusted for conscription age, birth year and conscription centre. Adjustment for maternal age and parental social factors (Model 2) increased the magnitude of the regression estimates, with highly significant associations for all outcomes (except for knee extension at birth order 3). Birth order showed inverse associations with BMI, knee extension strength and blood pressure, and positive associations with elbow flexion and hand grip strength.

**Table 2:** Regression coefficients for the effect of birth order on CVD risk factors with first-born as reference category.

	logBMI*100		Elbow flexion strength		Hand grip strength		Knee extension strength		SBP		DBP	
	*B*	CI	*B*	CI	*B*	CI	*B*	CI	*B*	CI	*B*	CI
Model 1												
2^nd^ born	−0.30***	−0.35, −0.25	4.15***	3.80,4.50	4.00***	3.60,4.41	1.62***	1.14,2.10	−0.15***	−0.19, −0.10	−0.09***	−0.13, −0.05
3^rd^ born	−0.05	−0.12,0.01	4.81***	4.34,5.28	3.10***	2.55,3.65	−1.78***	−2.42, −1.13	−0.25***	−0.31, −0.19	−0.14***	−0.19, −0.09
4^th^ born	0.22***	0.11,0.33	6.73***	6.00,7.46	2.48***	1.63,3.33	−5.23***	−6.21, −4.24	−0.20***	−0.29, −0.10	−0.13**	−0.21, −0.05
5^th^ born	0.09	−0.09,0.26	6.55***	5.40,7.70	3.09***	1.75,4.43	−9.68***	−11.21, −8.14	−0.11	−0.26,0.04	−0.16*	−0.28, −0.03
6^th^+born	0.22*	0.01,0.43	7.90***	6.55,9.24	2.38**	0.76,4.01	−14.78***	−16.59, −12.98	0.12	−0.06,0.30	0.06	−0.09,0.21
Model 2												
2^nd^ born	−0.39***	−0.44, −0.34	6.54***	6.17,6.92	6.02***	5.58,6.45	3.00***	2.49,3.51	−0.52***	−0.57, −0.48	−0.31***	−0.35, −0.27
3^rd^ born	−0.36***	−0.43, −0.28	8.49***	7.97,9.01	6.49***	5.88,7.10	1.30***	0.58,2.01	−0.95***	−1.02, −0.89	−0.54***	−0.60, −0.48
4^th^ born	−0.32***	−0.44, −0.20	10.81***	10.02,11.61	6.59***	5.67,7.52	−0.40	−1.47,0.67	−1.14***	−1.24, −1.04	−0.68***	−0.77, −0.59
5^th^ born	−0.63***	−0.82, −0.45	10.68***	9.47,11.88	7.60***	6.19,9.02	−3.45***	−5.06, −1.83	−1.21***	−1.37, −1.06	−0.83***	−0.96, −0.69
6^th^+born	−0.60***	−0.82, −0.37	12.43***	11.01,13.86	7.74***	6.02,9.47	−7.03***	−8.95, −5.11	−1.13***	−1.32, −0.95	−0.74***	−0.90, −0.58

*B*: Unstandardized regression coefficient; BMI, body mass index; DBP, diastolic blood pressure; SBP, systolic blood pressure; C, 95% confidence interval.

* *P*<0.05;

** *P*<0.01;

*** *P*<0.001.

Model 1: Adjusted for birth year, conscription age and conscription centre.

Model 2: Additionally adjusted for maternal age, fatheŕs and motheŕs social position and education.

Comparisons between fixed effects regression estimates in Table 3 (Model 4) with conventional linear regression estimates in Table 2 (Model 2) show substantial changes for most outcomes. In fixed effects models the associations are stronger for BMI and weaker for elbow flexion and SBP. Since fixed effects regression model removes the confounding influences of all fixed observed and unobserved genetic and social characteristics shared by the brothers including family size, we are now focusing mainly on Models 4, 5 and 6.

**Table 3:** Fixed effects regression coefficients (within family associations) for the effect of birth order on CVD risk factors.

	logBMI*100		Elbow flexion strength		Hand grip strength		Knee extension strength		SBP		DBP	
	*B*	CI	*B*	CI	*B*	CI	*B*	CI	*B*	CI	*B*	CI
Model 3												
2^nd^ born	−0.70***	−0.91,−0.49	1.67*	0.08,3.26	3.82***	2.02,5.62	−0.96	−3.12,1.22	−0.08	−0.29,0.13	0.14	−0.05,0.33
3^rd^ born	−0.91***	−1.29,−0.52	0.26	−2.58,3.11	2.04	−1.20,5.27	−4.43*	−8.29,−0.52	−0.14	−0.52,0.25	0.21	−0.13,0.55
4^th^ born	−0.93**	−1.51,−0.35	−0.49	−4.72,3.73	1.52	−3.31,6.35	−6.23*	−11.62,−0.11	−0.19	−0.76,0.37	0.05	−0.46,0.56
5^th^ born	−0.99*	−1.79,−0.19	−0.39	−6.19,5.40	3.88	−2.71,10.48	−8.20*	−15.48,0.33	−0.19	−0.97,0.59	−0.49	−1.18,0.21
6^th^+born	−0.84	−1.19,0.21	−4.28	−11.80,3.24	2.15	−6.44,10.74	−16.56**	−26.53,−6.13	−0.15	−1.16,0.86	−0.74	−1.64,0.16
Model 4												
2^nd^ born	−0.81***	−1.03,−0.57	1.53	−0.17,3.24	4.19***	2.25,6.13	−1.57	−3.91,0.76	−0.06	−0.29,0.17	−0.01	−0.22,0.19
3^rd^ born	−1.09***	−1.15,−0.68	−0.01	−3.02,3.00	2.52	−0.92,5.95	−5.55**	−9.69,−1.45	−0.11	−0.52,0.30	−0.05	−0.41,0.32
4^th^ born	−1.13***	−1.72,−0.53	−0.81	−5.18,3.55	2.04	−2.96,7.04	−7.53*	−13.17,−1.27	−0.16	−0.75,0.42	−0.23	−0.76,0.29
5^th^ born	−1.18**	−1.99,−0.37	−0.69	−6.58,5.21	4.44	−2.28,11.15	−9.44*	−16.91,−0.84	−0.15	−0.95,0.64	−0.76*	−1.46,−0.05
6^th^+born	−0.98	−2.03,0.07	−4.41	−11.98,3.15	2.77	−5.87,11.41	−17.33**	−27.44,−6.91	−0.11	−1.12,0.91	−0.95*	−1.85,−0.04
Model 5												
2^nd^ born	−0.83***	−1.06,−0.60	2.20*	0.51,3.90	6.25***	4.38,8.11	−0.77	−3.10,1.55	−0,00	−0,23;0,22	−0,01	−0,21;0,20
3^rd^ born	−1.13***	−1.54,−0.72	1.19	−1.81,4.18	6.20***	2.90,9.51	−4.13*	−8.24,−0.03	−0,01	−0,42;0,40	−0,03	−0,39;0,33
4^th^ born	−1.18***	−1.78,−0.58	0.73	−3.62,5.07	6.79**	1.99,11.60	−5.37	−11.30,0.56	−0,04	−0,62;0,55	−0,22	−0,74;0,31
5^th^ born	−1.23**	−2.04,−0.41	0.77	−5.09,6.64	8.94**	2.49,15.39	−7.12	−15.13,0.89	−0,03	−0,83;0,76	−0,74*	−1,45;−0,04
6^th^+born	−1.03	−2.08,0.02	−2.90	−10.43,4.63	7.43	−0.87,15.74	−15.36**	−25.57,−5.14	0,02	−1,00;1,03	−0,93*	−1,84;−0,02
Model 6												
2^nd^ born			3.83***	2.29,5.47	5.92***	4.03,7.80	1.36	−0.77,3.63	0.08	−0.14,0.31	0.05	−0.15,0.26
3^rd^ born			3.09*	0.37,5.99	4.86**	1.54,8.21	−1.60	−5.35,2.40	0.08	−0.32,0.48	0.05	−0.31,0.41
4^th^ born			2.39	−1.48,6.65	4.47	−0.31,9.41	−3.11	−8.46,2.75	0.04	−0.54,0.62	−0.14	−0.66,0.39
5^th^ born			2.67	−2.60,8.36	6.98*	0.56,13.59	−4.60	−11.87,3.27	0.06	−0.72,0.84	−0.66	−1.36,0.05
6^th^+born			−1.63	−8.43,5.67	4.88	−3.38,13.41	−13.61**	−22.91,−3.65	0.08	−0.92,1.07	−0.86	−1.76,0.04

*B*: Unstandardized regression coefficient; BMI, body mass index; DBP, diastolic blood pressure; SBP, systolic blood pressure; CI, 95% confidence interval.

* *P*<0.05;

** *P*<0.01;

*** *P*<0.001.

Model 3: Adjusted for birth year, conscription age and conscription centre.

Model 4: Additionally adjusted for maternal age.

Model 5: Model 4 additionally adjusted for height.

Model 6: Model 4 additionally adjusted for BMI.

Second and third born young men had 0.8% and 1.1% (p<0.001) lower BMI compared with first-born men (Model 4 and 5), but differences became weaker with higher birth order. Elbow flexion strength showed a significant association only after adjustment for height (p<0.05, Model 5) or BMI (p<0.001, Model 6), that is, once the effect of body size and mass were taken into account. The second born presented 2.20 N and 3.83 N more elbow flexion strength (after controlling for height and BMI, respectively) than the first-born. For hand grip strength, second-born men were approximately 6 N stronger than first-born men (6.25 N and 5.92 N adjusted for height and BMI, respectively) and the greatest difference from first-born men, 8.94 N, was observed for birth order 5 (when adjusted for height). Considering all three adjustments (Model 4, 5 and 6), knee extension strength showed the most robust association for birth order 6+, with 15.36 N/13.61 N (adjusted for height/BMI) less than the birth order 1. Finally, birth order showed non-significant association with SBP, and only a weak association with DBP (for birth order 5 and 6), which disappeared after adjustment for BMI.

As a sensitivity check, we re-estimated the results after excluding families with only one children from the analyses; the associations changed only little from those presented here (Table S1). The only detectable change was observed in DBP for second and third born (Model 3), but the statistical significance disappeared after adjusting for maternal age. We also repeated the analyses including birth weight as a covariate. Due to the lack of power, since birth weight was only available for a sub-population of 118 798 individuals, fixed effects regression models showed that all associations were non-significant. It must be noted, however, that even if non-significant, the results were very similar before and after adjustment for birth weight (results not shown).

## Discussion

During the last decades, major changes in population demography and family structure have occurred as consequence of the social and economic transition [35]. In this population-based cohort of more than one million young men, detailed information is presented on the association of birth order with several CVD risk factors, some of which have not been reported before. Our results revealed that in young adulthood, birth order effect is small on BMI and muscle strength, and non-apparent on blood pressure. First-borns seem to face more disadvantageous conditions of BMI, elbow flexion and hand grip strength, but more favourable level of knee extension strength.

The negative relationship detected between birth order and BMI in this population is in agreement with other studies carried out in young men of southern Brazil [13] and in young Bengali females [12]; however, positive [25] and non-significant [14], [26] associations have also been reported. Accordingly, first-born status has been related to elevated adiposity [11]–[13], and lower birth order showed to enhance the positive association between socioeconomic status and central adiposity in young adult Filipino males [36]. In these Swedish cohorts the greatest decrease was observed between birth order 1 and 2, and then BMI differences decreased by higher birth order. That is, first-born had 0.8% higher BMI than the second born and 1.2% higher than the fifth born. This indicates that although the effect is modest, the most disadvantageous position is the first-born. Our findings are in agreement with the tendency to post-natal catch-up growth observed in some first-born [19], which in turn has been associated with increased risk of obesity and higher adiposity in later life [20]–[23]. In the literature, although there is a trend towards disadvantageous conditions for firstborns, whether only child and firstborn status are differentially influenced remains largely unknown. In the present study, the exclusion of families with only one child from the analyses showed slightly weaker associations between birth order and BMI; however, Siervo et al. [13] detected that the exclusion magnified the effect. Celi et al. [37] observed no difference between being an only child or first-born in schoolchildren from Italy, and concluded that the status of firstborn is the aspect that proved to affect overweight or obesity. In contrast, two recent studies found that apart from the only child status, the last-born child presents an increased risk of overweight and obesity in Japanese and Danish schoolchildren [15], [16].

Muscle strength is an indicator of physical fitness, which is considered as one of the most important health markers nowadays [9], [38]. Skeletal muscle strength has been inversely associated with the incidence of CVD [5]–[8] and with increased risk of obesity, metabolic syndrome and all-cause and CVD mortality [9], [39], [40]. The role of muscular fitness in the prevention of diseases has become increasingly recognized [28]. However, to our knowledge, no study has analysed the influence of birth order on muscle strength. Although some studies carried out in this [41] and other populations [42]–[44] have shown that different muscle strengths are correlated among them, the present work suggests that birth order is differently associated with the three measures of muscle strength. The elbow flexion test showed unfavourable results for first-born young men with 3.83 N lower strength (body mass-independent) than the second born, although the effect diminished for men of higher birth order. Handgrip strength is widely used for assessing muscular fitness in epidemiological studies and has shown to be a strong predictor of morbidity and life expectancy [45]. In fact, even after arm muscle area and fat free mass were taken into account, hand grip strength was associated with incidence of CVD in men [8]. In this study we found that first-born men had approximately 6 N lower hand grip strength than second-born men when adjusted for body size, with smaller increases for men of higher birth order. The results from elbow flexion and handgrip strength tests and BMI suggest that first-born men are at increased CVD risk, although the difference with the second born is quite small. In contrast, knee extension strength showed the opposite direction, as well as a greater magnitude, with at least 13 N less for birth order 6+ than for first-born, implying that men of this high birth order are disadvantaged from this point of view.

Concerning the birth order effect on blood pressure, although the most common finding across studies indicate a negative association [10], [14], [17], non-significant [13] and J-shaped associations [27] have also been observed. As for BMI, rapid growth during infancy has been identified as an important determinant for blood pressure in adolescence [22] and adulthood [24]. Accordingly, some authors have proposed that an inverse relationship between birth order and blood pressure is established already in childhood [10] and adolescence [14]. However, in our study birth order differences in blood pressure were not apparent, which is in agreement with the study carried out in Brazilian men aged 17–19 years [13]. It should be noted that in these cohorts of Swedish men, the pattern differed somewhat between SBP and DBP. That is, whereas all birth orders presented similar association with SBP, for DBP, even if the statistical significance was only reached from birth order 5 (and disappeared after adjustment for BMI or excluding one child status), a negative trend was observed. Therefore, based on this tendency in young adulthood, we can speculate that these relationships may be more robust later in life, when higher and unhealthy blood pressure levels are most often observed. One explanation to the apparent lack of association with blood pressure in our sample is that birth order groups differ negligibly in height and BMI and thus present similar metabolic load. For a given metabolic load, a diminished metabolic capacity, which is predicted to be reduced by a low birth weight, increases blood pressure [46], [47]. In this sample, birth weight (available only for a sub-population) showed an increasing mean with each birth order, and the greatest difference was observed between the first and second born. However, even if blood pressure is sensitive to weight change and BMI, the similar height and BMI across birth order groups might have led to very small and non-significant differences in blood pressure.

This study has several strengths. The main advantage is the large sample size, which together with the analytical approach, allowed us to detect the within families variation with adequate power. In addition, this is the first study to investigate the long-term consequences of birth order on muscle strength. Since military conscription was mandatory during the study period, participation bias due to selection does not exist. But our study also has some limitations. First, our sample included only men and thus our results cannot be directly generalized to women. Second, the analysed sample was collected in young adulthood. This fact could be one of the reasons for the relatively small or non-significant birth order effect observed in this population, because more disadvantageous CVD risk factors levels tend to be observed in later life. Third, although military conscription was mandatory during the study period, disability or a severe chronic disease was a valid reason to be exempted, thus our cohort represents mainly healthy Swedish men at baseline. And finally, it should be mentioned that in the present study the association are significant because of a very large sample size, that is, in smaller samples some of the differences would not become statistically significant.

To summarize, the birth order effect on the analysed CVD risk factors in young adulthood is in general small and dependent on family characteristics. Birth order influence may vary in strength over time and place, but due to the unprecedentedly large population based dataset and that observed and unobserved characteristics shared by brothers were accounted for by fixed effect regression models, it is unlikely that birth order can have a substantially greater influence, at least in similar populations. Our findings indicate that birth order is inversely associated with BMI and knee extension, positively with elbow flexion and hand grip strength, and not associated with blood pressure. Since these associations may increase through adulthood, the birth order impact on CVD risk factors has public health implications because it can be used to target prevention and treatment strategies toward individuals at high risk. Finally, linking CVD risk factors with birth order suggests that part of the disadvantageous conditions observed in the populations could be attributed to the worldwide trend to smaller families and higher proportion of first-borns.

## Supporting Information

Table S1 **Fixed effects regression coefficients for the effect of birth order on CVD risk factors – families with only one child excluded.**
(DOCX)
