# Supplementary material for: Association of Birth Order with Cardiovascular Disease Risk Factors in Young Adulthood: A Study of One Million Swedish Men
Source: PLoS One. 2013 May 16;8(5):e63361. doi: 10.1371/journal.pone.0063361 (PMC3656047; doi:10.1371/journal.pone.0063361)
Supplement: Table S1 — Fixed effects regression coefficients for the effect of birth order on CVD risk factors – families with only one child excluded. (DOCX) [file pone.0063361.s001.docx]

**Table S1**. Fixed effects regression coefficients for the effect of birth order on CVD risk factors – families with only one child excluded

|  | logBMI*100 | |  | Elbow flexion strength | |  | Hand grip strength | |  | Knee extension strength | |  | SBP | |  | DBP | |
| --- | --- | --- | --- | --- | --- | --- | --- | --- | --- | --- | --- | --- | --- | --- | --- | --- | --- |
|  | *B* | CI |  | *B* | CI |  | *B* | CI |  | *B* | CI |  | *B* | CI |  | *B* | CI |
| Model 3 |  |  |  |  |  |  |  |  |  |  |  |  |  |  |  |  |  |
| 2^nd^ born | -0,66*** | -0,86;-0,45 |  | 1,79* | 0,23;3,35 |  | 3,83*** | 2,06;5,60 |  | -0,82 | -2,95;1,32 |  | -0,06 | -0,27;0,15 |  | 0,23* | 0,05;0,42 |
| 3^rd^ born | -0,87*** | -1,25;-0,49 |  | 0,19 | -2,63;3,01 |  | 1,80 | -1,41;5,01 |  | -4,55* | -8,41;-0,69 |  | -0,12 | -0,50;0,26 |  | 0,41* | 0,07;0,75 |
| 4^th^ born | -0,88** | -1,45;-0,31 |  | -0,76 | -4,96;3,43 |  | 0,98 | -3,83;5,79 |  | -5,97* | -11,70;-0,24 |  | -0,12 | -0,69;0,44 |  | 0,37 | -0,14;0,88 |
| 5^th^ born | -0,93* | -1,72;-0,15 |  | -0,62 | -6,36;5,12 |  | 3,31 | -3,23;9,85 |  | -7,98* | -15,83;-0,13 |  | -0,17 | -0,95;0,61 |  | -0,06 | -0,75;0,63 |
| 6^th^ + born | -0,78 | -1,81;0,24 |  | -4,29 | -11,73;3,15 |  | 1,29 | -7,23;9,81 |  | -16,76** | -26,89;-6,62 |  | -0,07 | -1,08;0,93 |  | -0,24 | -1,14;0,66 |
|  |  |  |  |  |  |  |  |  |  |  |  |  |  |  |  |  |  |
| Model 4 |  |  |  |  |  |  |  |  |  |  |  |  |  |  |  |  |  |
| 2^nd^ born | -0,75*** | -0,98;-0,53 |  | 1,63 | -0,05;3,31 |  | 4,20*** | 2,29;6,11 |  | -1,26 | -3,56;1,04 |  | -0,04 | -0,27;0,19 |  | 0,10 | -0,10;0,30 |
| 3^rd^ born | -1,04*** | -1,44;-0,64 |  | -0,12 | -3,11;2,88 |  | 2,30 | -1,12;5,72 |  | -5,43** | -9,53;-1,33 |  | -0,09 | -0,49;0,32 |  | 0,18 | -0,18;0,54 |
| 4^th^ born | -1,07*** | -1,66;-0,48 |  | -1,13 | -5,48;3,21 |  | 1,52 | -3,47;6,51 |  | -7,02* | -12,96;-1,08 |  | -0,08 | -0,67;0,51 |  | 0,11 | -0,42;0,64 |
| 5^th^ born | -1,12** | -1,92;-0,32 |  | -0,97 | -6,83;4,89 |  | 3,91 | -2,77;10,58 |  | -9,01* | -17,00;-1,01 |  | -0,12 | -0,92;0,67 |  | -0,31 | -1,02;0,39 |
| 6^th^ + born | -0,93 | -1,96;0,10 |  | -4,47 | -11,97;3,03 |  | 2,01 | -6,58;10,60 |  | -17,42** | -27,63;-7,21 |  | -0,02 | -1,04;0,99 |  | -0,44 | -1,34;0,47 |

*B*: Unstandardized regression coefficient; BMI, body mass index; DBP, diastolic blood pressure; SBP, systolic blood pressure; CI, 95% confidence interval.

**P* < 0.05; ***P* < 0.01; ****P* < 0.001.

Model 3: Adjusted for birth year, conscription age and conscription centre.

Model 4: Additionally adjusted for maternal age.
